# Correction: Cell type-specific long-range connections of basal forebrain circuit

**DOI:** 10.7554/eLife.22475

**Published:** 2016-10-24

**Authors:** Johnny Phong Do, Min Xu, Seung-Hee Lee, Wei-Cheng Chang, Siyu Zhang, Shinjae Chung, Tyler J Yung, Jiang Lan Fan, Kazunari Miyamichi, Liqun Luo, Yang Dan

Do JP, Xu M, Lee S-H, Chang W-C, Zhang S, Chung S, Yung TJ, Fan JL, Miyamichi K, Luo L, Dan Y. 2016. Cell type-specific long-range connections of basal forebrain circuit. *eLife* **5**:e13214. doi: 10.7554/eLife.13214.

Published 19, September 2016

In the published article, at the end of the 3rd paragraph of the Results section, the following statement should have been included “Another technical limitation of the study is that when the brain was removed for histological processing, the olfactory bulb was often damaged, which led to a significant underestimation of labeling (both the input neurons and axon projections) in the olfactory bulb.”

The article has been corrected accordingly.

